# Functional Characterization of Mechanosensitive Piezo1 Channels in Trigeminal and Somatic Nerves in a Neuron-on-Chip Model

**DOI:** 10.3390/ijms23031370

**Published:** 2022-01-25

**Authors:** Nikita Mikhailov, Lidiia Plotnikova, Prateek Singh, Rashid Giniatullin, Riikka H. Hämäläinen

**Affiliations:** 1 A. I. Virtanen Institute for Molecular Sciences, University of Eastern Finland, Neulaniementie 2, 70211 Kuopio, Finland; nikita.mikhailov@uef.fi (N.M.); lidiia.plotnikova@uef.fi (L.P.); 2 Finnadvance, Aapistie 1, 90220 Oulu, Finland; prateek@finnadvance.com

**Keywords:** migraine, trigeminal, dorsal root, mechanosensitive receptors, Piezo1, calcium imaging, microfluidics

## Abstract

Mechanosensitive ion channels, Piezo1 and 2, are activated by pressure and involved in diverse physiological functions, including senses of touch and pain, proprioception and many more. Understanding their function is important for elucidating the mechanosensitive mechanisms of a range of human diseases. Recently, Piezo channels were suggested to be contributors to migraine pain generation. Migraine is typically characterized by allodynia and mechanical hyperalgesia associated with the activation and sensitization of trigeminal ganglion (TG) nerve fibers. Notably, migraine specific medicines are ineffective for other types of pain, suggesting a distinct underlying mechanism. To address, in a straightforward manner, the specificity of the mechanosensitivity of trigeminal vs. somatic nerves, we compared the activity of Piezo1 channels in mouse TG neurons vs. dorsal root ganglia (DRG) neurons. We assessed the functional expression of Piezo1 receptors using a conventional live calcium imaging setup equipped with a multibarrel application system and utilizing a microfluidic chip-based setup. Surprisingly, the TG neurons, despite higher expression of the *Piezo1* gene, were less responsive to Piezo1 agonist Yoda1 than the DRG neurons. This difference was more prominent in the chip-based setup, suggesting that certain limitations of the conventional approach, such as turbulence, can be overcome by utilizing microfluidic devices with laminar solution flow.

## 1. Introduction

Mechanosensitive channels, Piezo1 and Piezo2, are non-selective Ca^2+^ permeable channels that transduce mechanical force into neuronal signals via transmembrane ion flux. Their functions range from sensing touch and pain to the control of plasticity in various tissues. Mechanosensation has been suggested to contribute to a variety of human disease conditions, including neuropathic pain, neurodegenerative diseases, trigeminal nerve injury, traumatic brain injury, cardiovascular disease and cancer [1,2,3,4,5].

Migraine pain likely originates from cranial meninges [6,7] which are innervated by trigeminal nerve fibers originating from the trigeminal ganglion (TG) neurons [8,9]. Migraine is characterized by pulsatile and throbbing pain, suggesting hypersensitivity to pulsating blood flow and, probably, to migraine-associated shear stress detected by mechanosensitive receptors [10]. Indeed, human TG neurons express mechanosensitive Piezo1 receptors [11] and we have recently demonstrated that the activation of Piezo1 receptors by a selective Piezo1 agonist Yoda1 [12] activates trigeminal neurons and induces meningeal trigeminal nociceptive signaling [13].

Both TG and dorsal root ganglia (DRG) are composed of primary sensory neurons. TG innervates the head whereas DRG send their processes to the rest of the body. The difference in the expression and activity of mechanosensitive receptors may give clues to their specific nociceptive functions in these ganglia [14]. To better understand the role of nociceptive mechanosensation and Piezo1 receptors role in pathophysiology of migraine, we wanted to compare the activity of Piezo1 receptors at the origin site of migraine pain (TG) with neurons located in the DRG and responsible for sensing somatic pain.

Thus, in this study, using live calcium imaging, quantitative PCR and immunolabelling, we investigated the difference in expression and function of Piezo1 receptors between TG and DRG.

Mechanosensitive receptors, and, in particular, Piezo1 receptors, can be activated by various stimuli that stretch the cell membrane either via changes in osmolarity or shear stress [10]. While control of osmolarity is easily achievable, controlling shear stress is more difficult and all conventional setups used to assess cell functions in vitro, i.e., live calcium imaging, encompass shear stress arising from solution changes and inability to achieve laminar stress-free conditions.

Thus, by taking advantage of modern microfluidic technology, that can provide more stable laminar-like fluid flow, which can be beneficial for studying mechanosensitive receptors, we conducted two independent sets of experiments, one performed in a conventional imaging setup, and another carried out in a microfluidic chip-based setup to compare function of TG and DRG neurons.

## 2. Results

### 2.1. Imaging of TG and DRG Neurons in Coverslips

**Figure 1:**
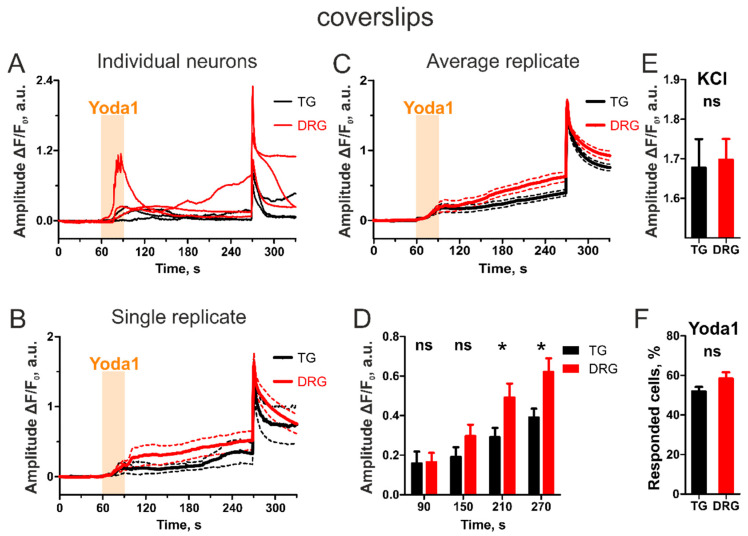
Yoda1-induced transients in TG vs. DRG neurons in a conventional calcium imaging setup utilizing fast application system. (**A**) Sample traces of individual TG and DRG neurons. (**B**) Average neuronal responses of TG and DRG cells obtained from single replicate coverslips. (**C**) Average neuronal responses of TG vs. DRG neurons (*n* = 9 coverslips). (**D**) A histogram of the results of unpaired Student’s *t*-test at different timepoints during Yoda1 washout. Significantly higher DRG responses are seen only after 2 min of washout (*n* = 9 coverslips, * *p* < 0.05 by unpaired Student’s *t*-test). (**E**) In contrast to Yoda1 responses, KCl evokes similar transients in both groups of neurons. (**F**) Dorsal root ganglion neurons tend to respond more frequently to Yoda1 than TG neurons. However, the difference is not significant.

We first conducted experiments utilizing a conventional calcium imaging setup equipped with the Rapid Solution Changer with cells seeded on glass coverslips (Figure 1). Exposure to the specific Piezo1 agonist Yoda1 (5 μM) induced transients in both TG and DRG neurons. Most of the responses were slow, which is typical for this agonist [13] and extended beyond application of Yoda1 (Figure 1A). Nine coverslips with cells from three different animals were analyzed for both TG and DRG. Results from a single coverslip (Figure 1B) and an average trace of all nine experiments (Figure 1C) are presented. The effect of Yoda1 is evident long after its withdrawal, during the entire washout recording. However, despite a clear trend of the DRG neurons to show a higher response than the TG neurons show, the difference becomes statistically significant only after two minutes of washout (*p* = 0.0292 after two minutes and *p* = 0.0128 after three minutes of washout, unpaired Student’s *t*-test). Notably, despite the constantly increasing difference between TG and DRG fluorescence, both neuron groups responded similarly to KCl application (*p* = 0.8212, by unpaired Student’s *t* test), verifying the difference in Yoda1 response. We further calculated the proportion of responding cells (=cells with more than 10% increase in fluorescence from baseline at the end of washout recording) and found a non-significant trend of the DRG cells to respond more frequently (52 ± 2% in TG vs. 58 ± 3% in DRG, *p* = 0.1105 by unpaired Student’s *t*-test). Notably, these results are in line with a previous study which demonstrated that 35% of TG neurons responded to 5 µM Yoda1 [13], compared with 52% of TG neurons responding to 25 µM Yoda1 in this study.

### 2.2. Imaging in Microfluidic Chips

**Figure 2:**
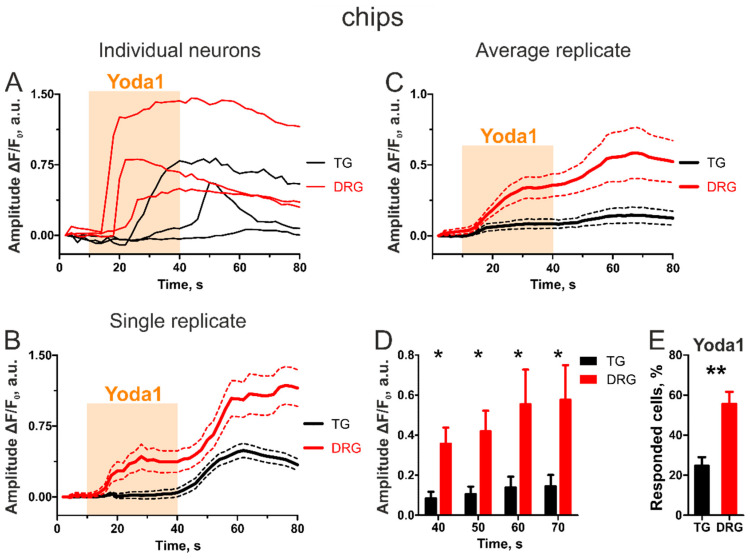
Yoda1-induced transients in TG vs. DRG neurons in microfluidic chips. (**A**) Sample traces of individual TG and DRG neurons. (**B**) Average neuronal responses of TG and DRG cells obtained from a single chip. (**C**) Average neuronal responses of all analyzed TG vs. DRG neurons (*n* = 10 chips). (**D**) In contrast to conventional imaging, in the chips a significant difference was seen already during Yoda1 application (*n* = 10 chips, * *p* < 0.05 by paired Student’s *t*-test). (**E**) Dorsal root ganglion neurons responded to Yoda1 significantly more frequently than TG neurons (*n* = 10 chips, ** *p* < 0.01 by paired Student’s *t*-test).

**Figure A1:**
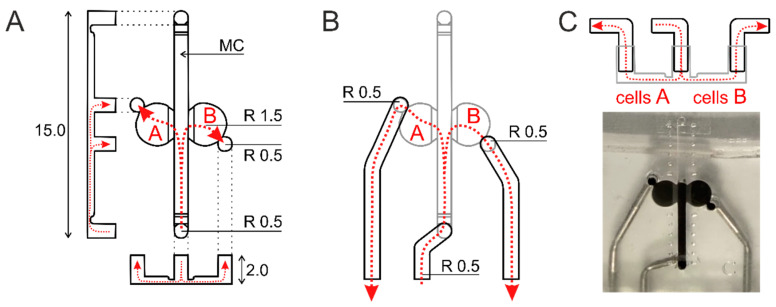
Microfluidic chip and application insert. (**A**) Blueprints of the microfluidic chip (cross sections and top view). (**B**) Top view blueprint of application insert–chip assembly. (**C**) Cross-section of application insert–chip assembly and top view photograph of the assembly.

**Figure A2:**
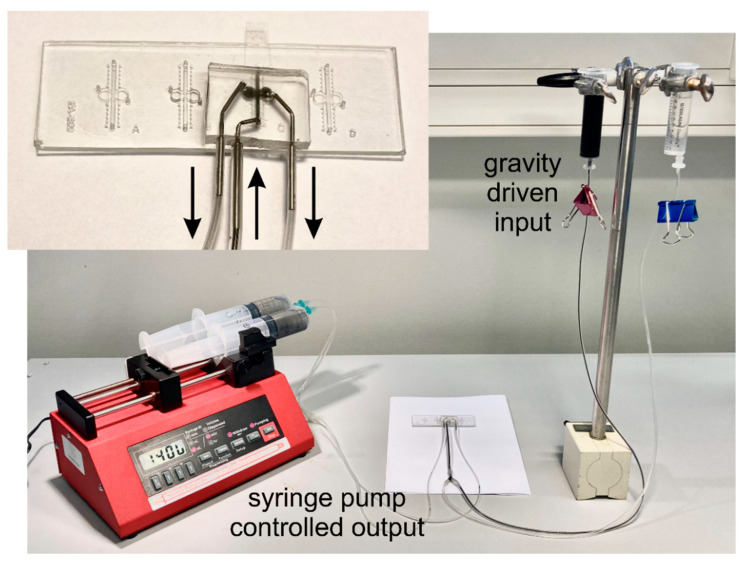
Chip-based setup. Solution input was driven by gravity, solution output was precisely controlled by microfluidic syringe pump with two syringes individually connected to two cell compartments. Close up panel demonstrates microfluidic chip in assembly with chip insert. Arrows show direction of solution flow.

Next, we analyzed the same TG and DRG neuronal responses to Yoda1 in a chip-based imaging setup (see Appendix A, Figure A1 and Figure A2 for details on chip design and setup). For both TG and DRG, a total of ten chip replicates with cells obtained from four different animals were analyzed. Similarly to the calcium signals obtained from the conventional setup, also in the microfluidic chips, the calcium transients in both TG and DRG neurons extended beyond Yoda1 application. Furthermore, the higher responsiveness to Yoda1 by DRG neurons was also similar to what was seen in the conventional setup (Figure 2A–C). However, this difference was more pronounced in the chip setup (Figure 2D). The difference between TG and DRG neurons was significant already at the end of the thirty second Yoda1 application (*p* = 0.0163 by paired Student’s *t*-test) and remained significant throughout the washout period after Yoda1 withdrawal. In addition, in contrast to imaging in the coverslips, the chip experiments demonstrated that the DRG neurons responded to Yoda1 significantly more frequently than the TG neurons did (Figure 2E, 25 ± 4% in TG vs. 56 ± 6% in DRG, *p* = 0.0039 by a paired Student’s *t*-test, responded cells = cells with more than 10% increase in fluorescence from baseline at the end of Yoda1 application).

Thus, the results obtained with the classical imaging setup and with the chip-based setup are similar, but the chip-based setup resulted in a sharper difference between the different neuron types and this difference was evident earlier than in the conventional set-up.

### 2.3. Turbulence in a Conventional Imaging Setup

A putative explanation for the chip-based setup resulting in a sharper difference could be the presence of turbulence in the conventional imaging setup. This turbulence, while probably neglectable in most calcium imaging experiments, becomes crucial when studying mechanosensitive receptors that are sensitive to shear stress.

**Figure 3:**
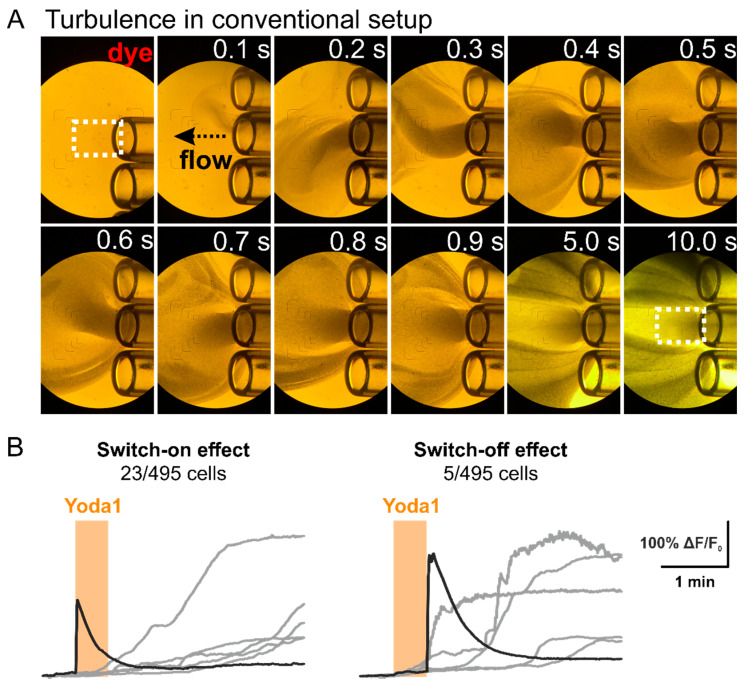
Turbulence occurring in conventional imaging setup. (**A**) Subsequent frames depict application of dye through fast application system. Tube switching causes substantial turbulence (0.2–0.6 s) that may underlie appearance of an atypical Yoda1 calcium transient. Black arrow indicates flow direction. (**B**) The sharp transients (black) different from normal Yoda1 responses (grey), seen in 5% of the cells (23/495) in the beginning of the application and in 1% of the cells (5/495) in the end of the application are likely associated with this non-intentional shear stress. White dashed rectangles mark target imaging region.

To visualize the turbulence, we imitated solution exchange using water as the initial solution and suspension of visible dye particles as the second substitute solution (Figure 3A). Two types of unwanted behavior were spotted. Firstly, acute turbulence occurred at the time of solution exchange. This was associated with a rotation of the application capillaries (approximately 1 s). Secondly, a persistent turbulence was seen continuously throughout the experiment after equilibration of the system at the edges of the imaging zone.

**Figure A3:**
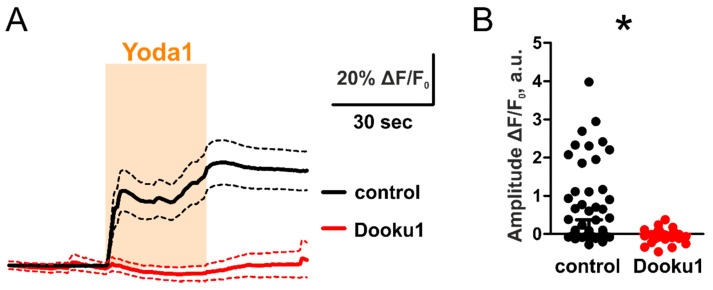
Dooku1 diminishes Yoda1-induced transients. (**A**) Traces depict average fluorescence of control and Dooku1 treated neurons (*n* = 88 neurons for control and 29 for Dooku1-treated group, presented as mean ± SEM). (**B**) Neurons treated with Dooku1 demonstrated significantly lower responses at the end of the 30 s Yoda1 application (* *p* < 0.05 by Mann–Whitney U test, data presented as median with interquartile range).

Acute turbulence causes shear stress, which is considered to activate mechanosensitive receptors [10]. Indeed, in our experiments a fraction of neurons (~5%, 23/495 cells) responded acutely with sharp calcium transients presenting immediately at the beginning of the Yoda1 application (Figure 3B, switch-on effect, trace in black). This type of response is not typical for Yoda1-induced transients, which are typically slow and delayed (Figure 3B, traces in grey). In addition, a small proportion of neurons (~1%, 5/495 cells) responded at the beginning of the washout after withdrawal of Yoda1 (Figure 3B, switch-off effect, trace in black). A selective antagonist of Piezo1 receptors, Dooku1, demonstrated ability to diminish both acute and slow Yoda1-induced responses, thus verifying that the responses are specific to Piezo1 activation (Appendix B, Figure A3).

### 2.4. Chip Setup Provides Uniform Flow and Smooth Solution Transitions

**Figure 4:**
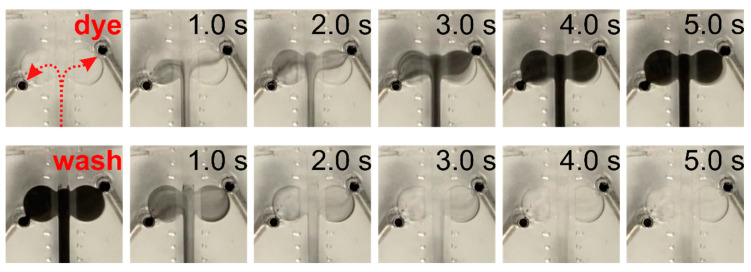
In the chip setup application insert assembly provides gradual turbulence-free substitution of liquid (dye application depicted, red arrows indicate flow direction). For details of the chip design and chip-based setup see Appendix A, Figure A1 and Figure A2.

We further tested the presence of turbulence in the chip-based setup, where smoother and turbulence-free transition of fluids was seen (Figure 4). However, the transition from one solution to another took longer in the chip setup than in the conventional setup (5 s in the chip vs. 1 s in the conventional setup).

### 2.5. Piezo1 Expression in TG Cells Is Higher Than in DRG Cells

**Figure 5:**
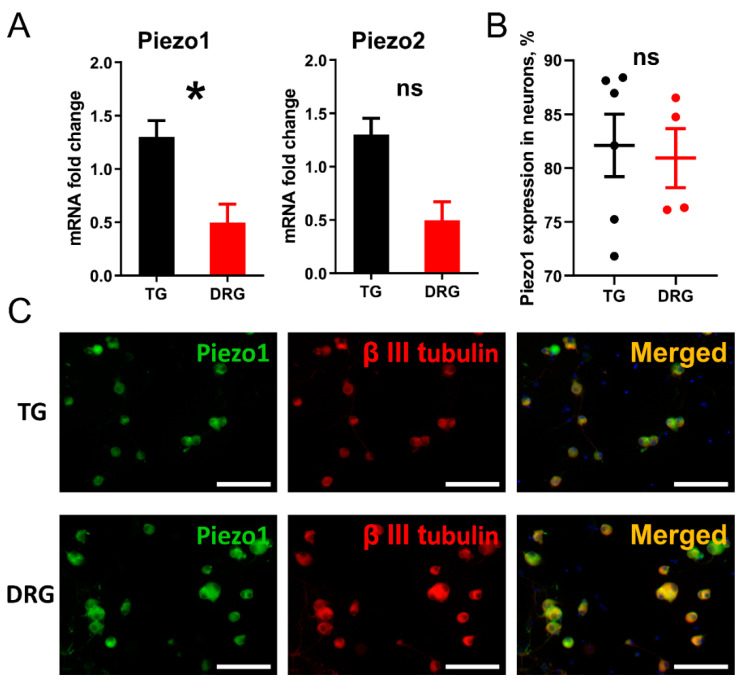
(**A**) Expression of *Piezo1* and *Piezo2-*coding mRNA in TG vs. DRG cells. Data is normalized against *18S* mRNA level. * *p* < 0.05 by paired Student’s *t*-test (*n* = 3 biological replicates, data presented as mean ± SEM). (**B**) Expression rate of Piezo1 protein in TG vs. DRG neurons (co-expression with β III tubulin), data presented as mean ± SEM. (**C**) Immunocytochemical staining of Piezo1 (green) and β III tubulin (red) in TG vs. DRG neurons. Blue colour—DAPI. Scale bars—100 μm.

Finally, we analyzed the expression of *Piezo1* and *Piezo2* coding mRNAs in DRG and TG cultures by qPCR (Figure 5A). In contrast to the higher activity of Piezo1 in DRG cells than in TG cells, the *Piezo1* gene expression was significantly higher in TG cells than in DRG cells. A similar, however, not significant trend was found for the *Piezo2* gene expression.

Next, we conducted immunocytochemical staining of Piezo1 protein in TG vs. DRG cells (Figure 5B,C). The cells were also stained for β III tubulin as a neuronal marker. In total six coverslips with TG cells from three different animals and four coverslips with DRG cells from 2 different animals were used for analysis. Co-expression of Piezo1 with β III tubulin was quantified in β III tubulin positive cells (Figure 5B), and no difference was detected between TG and DRG neurons (82 ± 3% in TG and 81 ± 3% in DRG).

## 3. Discussion

Here, we provide, for the first time, a microfluid chip system for a comparative study of mechanosensitive receptor activity in two samples of sensory neurons originating from different sources. Similar setups can also be used for studying other signalling receptors and may have broad applications for biomedical studies. Multibarrel application systems are traditionally used in patch-clamp or live imaging experiments to provide fast exposure (in 10-100 ms range) of the agonists to the respective receptors in cultured cells. However, the beneficial property of a fast application system also has adverse effects, which are particularly important when detecting mechanosensitive currents. A substantial turbulence occurs during solution exchange, caused by the fast rotation of tubes, and a continuous presence of turbulence at the edges of the targeted imaging zone is seen throughout experiments. Although for most live imaging experiments these features are not significant, they can cause unwanted responses when studying cells that express mechanosensitive receptors. The shear stress caused by rotation of tubes during solution change can directly activate mechanosensitive receptors in all cells at the imaging zone at the time of solution exchange. Turbulence at the edge of the imaging zone is present throughout the experiment and affects mostly cells located outside of the visual field, however, as signals can be transmitted through neuronal connections, also this can occasionally lead to secondary responses in the cells inside the imaging zone.

In this study, we investigated the effect of Yoda1, an agonist of the mechanosensitive Piezo1 receptor, on neuronal cultures derived from TG and DRG of 3-5 weeks old mice. Typically, Yoda1 induces slow and long-lasting responses that often develop after Yoda1 withdrawal. However, when applied trough the rapid solution changer, a proportion (5%) of the cells responded immediately at the time of the Yoda1 application, and even more interestingly, a small fraction of the cells (1%) responded right after Yoda1 withdrawal. These responses are apparently associated with shear stress due to visible solution exchange in our experiments with application of the dye. Interestingly, while the switch on effects are common for all studies, we have not seen withdrawal induced responses in experiments without Yoda1 [15]. Thus, a plausible explanation for these fast responses at the edges of Yoda1 application is a combined effect of the shear stress together with the effect of Yoda1, which is known not only to be a direct agonist of Piezo1 receptors, but also as a co-agonist facilitating force-induced activation of Piezo1 receptors [16].

These unwanted stress-induced transients can affect the outcome of experiments on mechanosensitive receptors, masking the true effect of the studied compound and resulting in noise that dampens the putative differences between experimental groups. In the current study, we tested the effect of Yoda1 in TG vs. DRG neurons in two independent setups—conventional imaging setup equipped by RSC-200 and a microfluidic chip-based setup.

The results obtained with these two approaches are very similar. In both setups the TG neurons responded less frequently to the application of Yoda1 and the intensity of the response was weaker than in DRG cells. However, the chip-based setup provided sharper results with higher statistical power both in the magnitude and the frequency of the neuronal responses. While conventional imaging provided significant difference only at the latter stage of the five-minute-long washout, in the microfluidic chips a significant difference was detected much earlier, within thirty seconds of the Yoda1 application. In both approaches, the DRG neurons tended to respond more frequently than the TG neurons; however, this difference was only significant in the chips. Interestingly, the number of responsive cells in the conventional imaging setup was higher than in the chip setup for TG (52% in conventional imaging vs. 25% in chips) neurons but at the same level for DRG neurons (58% in conventional imaging vs. 56% in chips). This may be due to the additional transient-inducing factors in the conventional setup, shear stress and turbulence, having more profound effect on TG neurons, which had a reduced response to Yoda1, than on DRG neurons that are highly activated by Yoda1 and thus the additional shear stress does not seem to further increase the response.

The obtained results are interesting for migraine. Previous studies have suggested the involvement of mechanosensitive receptors in migraine generation [10,13]; however, the migraine-related TG neurons demonstrated lower excitability to Piezo1 agonist Yoda1 than DRG neurons, suggesting that there are fewer active Piezo1 channels incorporated into the cellular membrane in TG than in DRG neurons. However, the *Piezo1* mRNA showed substantial expression in TG neurons, even higher than in DRG neurons. Further, while, substantial variation of Piezo1 protein expression was seen between individual neurons, no significant difference was detected in the overall protein expression between TG and DRG neurons. These data could indicate that while the mechanosensitive activity in TG is not high at normal physiological conditions, there is a reserve capacity for increased sensitivity, which may be activated in migraine or other pathogenic conditions. Other studies have also reported higher expression of mechanosensitive channel genes (*ASIC1*) in TG than in DRG, further supporting high capacity of the TG neurons to mechanosensitive responses [14]. However, as our experiments were conducted on mixtures of neuronal and glial cells, the *Piezo1* expression results are not fully transferrable to exact expression rate in neurons.

As Piezo1 and mechanosensation have many additional functions besides migraine generation, the higher activity of Piezo1 in DRG than in TG neurons, is not surprising. Similar results of differences in activity of nociceptive factors in TG vs. DRG neurons have previously been reported for CGRP (calcitonin gene-related peptide), the key migraine mediator [14,17]. It is well established that CGRP has pro-nociceptive effects in migraine, sensitizes TG neurons [18] and induces migraine attacks [19]. The levels of CGRP are increased in chronic migraineurs [20], and attenuation of CGRP has benefits in migraine treatment [21], thus the importance of CGRP in migraine generation is undisputable. Nevertheless, comparison of TG and DRG [14] demonstrated lower expression of both the *CGRP* gene and CGRP protein in TG neurons than in DRG neurons.

Our study was conducted on primary cells derived from migraine-free animals. Further studies are needed to compare excitability of naïve TG neurons either with TG neurons derived from transgenic migraine mice, such as familial hemiplegic migraine model, or with neurons with induced migraine state (such as neurons treated with CGRP), to verify whether this increased sensitivity indeed underlies origin of migraine.

## 4. Materials and Methods

### 4.1. Animals and Cell Culture

For our study, 3–7 weeks old male C57BL/6JOlaHsd mice were provided by Lab Animal Center of the University of Eastern Finland. All procedures with animals were in accordance with the European Community Council Directive of 22 September 2010 (2010/63/EU). Protocols were approved by the Animal Care and Use Committee of the University of Eastern Finland (license EKS-008-2019).

Primary culture from TG and DRG were prepared as described elsewhere [22,23]. Briefly, after isolation TG and DRG were transferred to 0.5 mL of enzymatic cocktail (trypsin 0.5 mg/mL, collagenase 1 mg/mL, DNAse 0.2 mg/mL, all Sigma-Aldrich, St. Louis, MI, USA) and incubated in thermomixer (Eppendorf, Hamburg, Germany) at 37 °C at 1000 RPM. After dissociation, cells were filtered with a 100 µm cell strainer and resuspended in 100 µL (TG) or 50 µL (DRG) of media. Then, the cells were either plated on glass coverslips, or introduced into microfluidic chips (both coated with 0.2 mg/mL PLL for 1 h). The microfluidic chips had two compartments for simultaneous cultivation of both TG and DRG neurons in one chip (for details on cell culture in chips see Appendix A). After initial introduction, the cells were left to attach for 2 h, and then chips were filled with media and coverslips were transferred to 35 mm Petri dishes filled with 2 mL of media. Cells were used for experiments after 1 day in vitro.

### 4.2. Microfluidic Chips and Chip-Insert

Microfluidic chips (Brain Disorder (BD) chip, Finnadvance, Oulu, Finland) made of polydimethylsiloxane (PDMS) slab, bearing all desired features (channels and cell compartments) sealed to a standard microscopic glass as described elsewhere [24] were used in the study.

The in-house built application insert was produced from PDMS with embedded metal tubes. Tubes’ outlets were positioned against the respective inlets in BD microfluidic chip allowing hermetical BD chip-application insert assembly. Detailed description of BD microfluidic chip and application insert are in the Appendix A.

### 4.3. Fluo-4 AM Imaging in Conventional Imaging Setup

Cells were incubated in Fluo-4 AM solution (1×, Fluo-4 Direct Calcium Assay Kit, Invitrogen, Waltham, MA, USA) for 30 min at 37 °C. Then, coverslips were post-incubated for 10 min at 37 °C in basic salt solution (pH 7.4) containing (in mM):152 NaCl, 10 HEPES, 10 glucose, 2.5 KCl, 2 CaCl_2_, 1 MgCl_2_.

Imaging setup (TILL Photonics imaging system, TILL Photonics, Kaufbeuren, Germany) was equipped with Rapid Solution Changer (RSC-200, BioLogic Science Instruments, Seyssinet-Pariset, France). Images were acquired with CCD camera (SenciCam, PCO Imaging, Kelheim, Germany) at 10× magnification on Olympus IX-70 microscope (Tokyo, Japan) at 2 FPS.

The imaging protocol included a 1 minute baseline (BSS), 30 s of 25 µM Yoda1, 3 min of washout (BSS) and 2 s of 30 mM KCl application (with compensated osmolarity, as a marker of neurons).

Data was acquired with Live Acquisition and analyzed with Offline Analysis software (TILL Photonics, Kaufbeuren, Germany).

### 4.4. Live Calcium Imaging in Chip-Based Setup

Cells were incubated in Fluo-4 AM for 1 h and post-incubated in BSS for 10 min (both at 37 °C). Then, the chips were transferred to an Axio Imager M2 microscope (Zeiss, Oberkochen, Germany). Using the application, insert chips were connected to reservoirs with BSS and Yoda1 solution, and to syringe pump (New Era Pump Systems, Farmingdale, NY, USA) equipped with two 50 mL syringes. Solution input was gravitation-driven, solution output was controlled by syringe pump at rate of 1400 µL/min through each of the cell compartments.

The imaging protocol included 10 s baseline (BSS), 30 s of 25 µM Yoda1 and 40 s of washout.

Cells were imaged at 0.5 FPS with ZEN 2 software (Zeiss, Oberkochen, Germany) and analyzed by ImageJ 1.47v (National Institute of Health, Bethesda, MD, USA).

### 4.5. mRNA Expression of Piezo Genes

Total RNA was isolated from the TG and DRG cell cultures using the RNeasy Mini kit (Qiagen, Hilden, Germany) according to the manufacturer’s protocol. The extracted RNA was DNAse-treated and eluted in nuclease-free water. RNA was quantified using the NanoDrop ND-1000 spectrophotometer (ThermoFisher Scientific, Waltham, Massachusetts, USA). cDNA (2.5 ng/μL) was synthesized using 500 ng of total RNA, dNTP, random hexamer primer, Maxima reverse transcriptase in the presence of ribonuclease inhibitor (all reagents ThermoFisher Scientific). The relative expression levels of mRNAs encoding the selected genes were analyzed in triplicates and measured by RT-qPCR (StepOnePlus Real-Time PCR System, ThermoFisher Scientific, Waltham, MA, USA) using SYBRgreen assay (Maxima SYBR green master mix, ThermoFisher Scientific, Waltham, MA, USA). The primer sequences were as follows: Piezo1-F 5′-TCATCATCCTTAACCACATGGTG-3′; Piezo1-R 5′-TGAAGACGATAGCTGTCATCCA-3′; Piezo2-F 5′-TGATTCATGCCTGTTGGTTG-3′; Piezo2-R 5′-TGAAATCCGGGAAGTACAGC-3′; 18S-F 5′-CGCCGCTAGAGGTGAAATTC-3′; 18S-R 5′-CGAACCTCCGACTTTCGTTCT-3′ (all primers Invitrogen, Waltham, MA, USA). The *Piezo* results were normalized to the levels of endogenous control, *18S* rRNA. Relative mRNA expression was calculated with the comparative 2^−∆∆Ct^ method, in which Ct is the threshold cycle number and results presented as relative fold change to one of the TG samples.

### 4.6. Immunocytochemical Staining of Piezo1 in TG vs. DRG Neurons

A culture of TG and DRG neurons on glass coverslips was used for the staining. Coverslips were placed in 24 well plate. Then, cells underwent following steps: fixation in 4% PFA for 20 min, permeabilization in 0.2% Triton X-100 for 10 min, unspecific binding sites blockage in 10% normal goat serum for 30 min, blockage with Mouse-on-Mouse IgG Blocking Solution (Invitrogen, Waltham, MA, USA) for 30 min. Then, cells were incubated overnight at +4 °C with the primary antibodies, washed with PBS and incubated in secondary antibodies for 2 h at room temperature.

As primary antibodies, we used MA5-32876 (Invitrogen, Waltham, MA, USA), mouse, 1:150 dilution for Piezo1 receptors; ab107216 (Abcam, Cambridge, UK), chicken, 1:1000 dilution for β 3 tubulin, as a marker of neurons.

As secondary antibodies, we used Alexa Fluor 488 Goat anti-mouse (1:1000 dilution) to bind Piezo1 primary antibodies and Alexa Fluor 568 Goat anti-chicken (1:1000 dilution) to bind β 3 tubulin primary antibodies (both Invitrogen).

Axio Imager M2 microscope (Zeiss, Oberkochen, Germany) was used to image cells. Data were analyzed by ImageJ 1.47v (National Institute of Health, Bethesda, MD, USA).

## 5. Conclusions

Our comparative study on Piezo1 expression in TG vs. DRG demonstrates that, despite the proven role of Piezo1 receptors in trigeminal nociception, TG neurons have less functional Piezo1 receptors than DRG neurons. Nevertheless, as the expression of Piezo-coding mRNA is higher in TG than in DRG, and the protein is expressed at similar levels in both types of neurons, the TG neurons seem to harbor a reserve capacity, which can acutely increase Piezo1 functionality upon need, and which may also be activated in migraine conditions.

In addition, we show that studies on cells expressing mechanosensitive receptors require special care to avoid unwanted and non-specific activation of mechanosensitive receptors. Microfluidic chips recommend themselves as optimal platform for these types of studies, as the laminar liquid flow can be precisely controlled in them.

We used commercially available microfluidic chips in this study. However, the possibilities of microfluidic device production permits the standard design to be easily changed into a custom one, that will optimally fit the needs of a particular live calcium imaging experiment.

Microfluidic devices have multiple additional benefits. They can provide precisely controlled and highly reproducible experimental conditions. In addition, due to their small scale, experiments with microfluidic devices require much smaller sample volumes (in our study—3.5 µL for chips vs. 30 µL for coverslips). This can play a critical role when working with primary cultures and restricted sample sizes and cell numbers. Similarly, much less of the various reagents are needed, which can be beneficial when testing expensive substances.

## Data Availability

All data supporting the conclusions are available upon request from the corresponding authors.

## Appendix A. Microfluidic Chip-Based Setup

The microfluidic chips (Finnadvance, Finland) had two compartments (A and B, Figure A1A), with an individual inlet/outlet in each of them. This facilitated a simultaneous cultivation of two different cell cultures. For this, we proceeded as follows: TG and DRG cell suspensions were introduced in PLL-covered cell compartments, one cell suspension to compartment A, another to compartment B. Volume of introduced cell suspension (appx. 3.5 µL) was enough to completely fill cell compartments, leaving at the same time middle channel (MC on Figure A1A) free of cells. After two hours, when cells had attached, chips were washed by introducing media through the middle channel and unattached cells were removed leaving two different cell types growing separately in the two compartments of the chip.

To provide precise control of flow inside the microfluidic chips we designed an in-lab chip insert. Figure A1 depicts blueprints of the microfluidic chip and the chip insert.

Figure A2 demonstrates main principles of chip-based setup—gravitation-driven input, syringe pump-controlled output and chip-application insert assembly.

## Appendix B. Piezo1 Selective Antagonist, Dooku1, Diminishes Yoda1 Induced Responses

To validate the specificity of the Yoda1 effect, we tested Yoda1 action in TG neurons under application of Dooku1, a selective antagonist of Piezo1 receptors. Cells were pre-incubated with 10 µM Dooku1 for 40 min prior to experiment. In addition, 10 µM Dooku1 was added to all solutions applied during analysis.

Dooku1 demonstrated ability to diminish Yoda1-induced calcium transients in TG neurons and verified that the responses were specific to Piezo1 (* *p* < 0.05, by Mann–Whitney U test, Figure A3).
